# Supplementary material for: Illuminating the Plant Rhabdovirus Landscape through Metatranscriptomics Data
Source: Viruses. 2021 Jul 5;13(7):1304. doi: 10.3390/v13071304 (PMC8310260; doi:10.3390/v13071304)
Supplement: Supplementary file 1 [file viruses-13-01304-s001.zip › viruses-1243763-supplementary.pdf]

viruses

MDPI

Supplementary Information

Illuminating the Plant Rhabdovirus Landscape through Metatranscriptomics Data

Nicolás Bejerman [1,2,*], Ralf G. Dietzgen [3] and Humberto Debat [1,2]

1 Instituto de Patología Vegetal, Centro de Investigaciones Agropecuarias, Instituto Nacional de Tecnología Agropecuaria (IPAVE—CIAP—INTA), Camino 60 Cuadras Km 5.5, Córdoba X5020ICA, Argentina; debat.humberto@inta.gob.ar
2 Consejo Nacional de Investigaciones Científicas y Técnicas, Unidad de Fitopatología y Modelización Agrícola, Camino 60 Cuadras Km 5.5, Córdoba X5020ICA, Argentina
3 Queensland Alliance for Agriculture and Food Innovation, The University of Queensland, St. Lucia, Brisbane, QLD 4072, Australia; r.dietzgen@uq.edu.au
* Correspondence: bejerman.nicolas@inta.gob.ar

Citation: Bejerman, N.; Dietzgen, R.G.; Debat, H. Illuminating the Plant Rhabdovirus Landscape through Metatranscriptomics Data. *Viruses* **2021**, *13*, x. https://doi.org/10.3390/v13071304

Academic Editor: Karl Andrew White

Received: 17 May 2021
Accepted: 26 June 2021
Published: 5 July 2021

Publisher's Note: MDPI stays neutral with regard to jurisdictional claims in published maps and institutional affiliations.

Copyright: © 2021 by the authors. Licensee MDPI, Basel, Switzerland. This article is an open access article distributed under the terms and conditions of the Creative Commons Attribution (CC BY) license (http://creativecommons.org/licenses/by/4.0/).

Table S1. Virus names, abbreviations and National Center for Biotechnology Information (NCBI) accession numbers of plant rhabdovirus sequences used in this study.

Virus name	Abbreviation	Accession number
alfalfa-associated nucleorhabdovirus	AaNV	MG948563
alfalfa dwarf virus	ADV	KP205452
Alopecurus myosuroides varicosavirus 1	AMVV1	LN713933; LN713934
apple rootstock virus A	ApRVA	MH778545
Bacopa monnieri virus 1	BmV1	BK014479
Bacopa monnieri virus 2	BmV2	BK014480
barley yellow striate mosaic virus	BYSMV	KM213865
bean associated rhabdovirus	BaCV	MK202584
bird's-foot trefoil virus 1	BFTaV	BK010826
black currant-associated rhabdovirus	BCaRV	MF543022
cabbage cytorhabdovirus 1	CCyV1	KY810772
cardamom vein clearing virus 1	CdVCV1	MN273311
citrus-associated rhabdovirus	CiaRV	MT302547
citrus chlorotic spot virus	CiCSV	KY700685; KY700686
citrus leprosis virus N	CiLV-N	KX982176; KX982179
clerodendrum chlorotic spot virus	ClSCV	MG938506: MG938507
chrysanthemum yellow dwarf-associ-ated virus	ChYDaV	MW039593
coffee ringspot virus	CoRSV	KF812525; KF812526
colocasia bobone disease-associated vi-rus	CBDaV	KT381973
constricta yellow dwarf virus	CYDV	KY549567
cucurbit cytorhabdovirus 1	CuCV1	MT381995
datura yellow vein virus	DYVV	KM823531
eggplant mottle dwarf virus	EMDV	KJ082087
green Sichuan pepper nucleo-rhabdovirus	GSPNuV	MH323437
Iranian citrus ringspot-associated virus	IrCRSaV	KP255975
joa yellow blotch-associated virus	JYBaV	MW014292
Kenyan potato cytorhabdovirus	KePCyV	MN689395
lettuce big-vein associated virus	LBVaV	AB075039; AB114138
lettuce necrotic yellows virus	LNYV	AJ867584
lettuce yellow mottle virus	LYMoV	EF687738
maize associated cytorhabdovirus	MaCyV	KY965147
maize fine streak virus	MFSV	AY618417
maize Iranian mosaic virus	MIMV	MF102281
maize mosaic virus	MMV	MK828539
Morogoro maize-associated virus	MMaV	MK112501
maize yellow striate virus	MYSV	KY884303
northern cereal mosaic virus	NCMV	MH282832
orchid fleck virus	OFV	LC222629; LC222630
papaya virus E	PpVE	MK202584
paper mulberry mosaic-associated vi-rus	PMuMaV	MN872813
peach virus 1	PeV1	MN520414

persimmon virus A	PeVA	AB735628
Physostegia chlorotic mottle virus	PhCMoV	KY859866
potato yellow dwarf virus	PYDV	GU734660
raspberry vein chlorosis virus	RVCV	MK257717
red clover-associated varicosavirus	RCaVV	MF918568; MF918569
rice stripe mosaic virus	RSMV	MH720469
rice yellow stunt virus	RYSV	AB011257
rose virus R	RVR	MT952336
sonchus yellow net virus	SYNV	L32603
sowthistle yellow vein virus	SYVV	MT185675
strawberry crinkle virus	SCV	MH129615
strawberry virus 1	StrV1	MK211270
taro vein chlorosis virus	TaVCV	AY674964
tomato yellow mottle-associated virus	TYMaV	KY075646
Trichosanthes-associated rhabdovirus 1	TrARV1	BK011194
Trifolium pratense virus A	TpVA	MH982250
Trifolium pratense virus B	TpVB	MH982249
wheat yellow striate virus	WYSV	MG604920
Wuhan insect virus 4	WhIV4	KM817650
Wuhan insect virus 5	WhIV5	KM817651
Wuhan insect virus 6	WhIV6	KM817652
yerba mate chlorosis-associated virus	YmCaV	KY366322
yerba mate virus A	YmVA	MN781667

Table S2. Summary of assembly statistics of the plant rhabdoviruses sequences identified from the transcriptome data available in the NCBI database.

Virus name	Abbreviation	Bioproject ID	SRA Accesion	Total virus reads	Mean Coverage	Reads per Millon
Agave tequilana virus 1	ATV1	PRJNA193469	SRR789743	26874	306.54X	297.20
Asclepias syriaca virus 2	AscSyV2	PRJNA210776	SRR5117431	21120	164.84X	439.21
Cuscuta reflexa virus 1	CusReV1	PRJNA290291	SRR2142348	119	6.88X	63.99
Persicaria minor virus 1	PerMiV1	PRJNA208436	SRR917962	885	97.12X	1472.05
Plectranthus aromaticus virus 1	PleArV1	PRJNA491230	SRR7896533	17676	156.43X	2813.02
Rhododendron delavayi virus 1	RhoDeV1	PRJNA358123	SRR5121284	7841	51.43X	98.91
Allium chinense virus 1	AChV1	PRJNA310810	SRR3144560	878	13.75X	7.86
Anthurium amnícola virus 1	AntAmV1	PRJNA288827	SRR2089239	51637	620.63X	1495.45
Asclepias syriaca virus 1	AscSyV1	PRJNA210776	SRR5117431	51339	387.18X	1067.65
Bemisia tabaci -associated virus 1	BeTaV1	PRJNA237273	SRR1159208	88609	687.10X	1057.02
Dioscorea composita virus 1	DiCoV1	PRJNA253902	SRP044768	19538	196.18X	40.64

Glehnia littoralis virus 1	GlLV1	PRJNA248158	SRP042106	11812	97.84X	110.35
Gymnadenia densiflora virus 1	GymDenV1	PRJNA504609	SRR8175725	29407	301.22X	593.66
Lotus corniculatus virus 1	LotCorV1	PRJNA77207	SRR364671	3535	25.06X	132.41
Nymphaea alba virus 1	NymAV1	PRJNA472003	SRR7224571	135173	1048.99X	398.62
Pelargonium radula virus 1	PelRaV1	PRJNA491235	SRR7900215	676	6.07X	46.94
Suaeda salsa virus 1	SuSV1	PRJNA395283	SRP113741	520	8.67X	1.33

Table S3. Characteristics of deduced proteins encoded by each assembled rhabdovirus sequence determined by predictive algorithms.

Virus	RNA	ORF No[a]	Gene name	Mr (kDa)	IEP	TMHMM	Signal P cleavage site	Predicted NLS [cNLS Mapper score] #	Predicted NES	Highest scoring virus- protein/*E*-value/query coverage% (Blast P)
Agave tequilana virus 1	1	1<br>2<br>3<br>4<br>5<br>6	N<br>P<br>P3<br>M<br>G<br>L	50<br>37.4<br>32<br>28.8<br>67.8<br>221.2	8.49<br>5.58<br>8.54<br>6.71<br>6.45<br>6.97	-<br>-<br>-<br>-<br>551–573<br>-	-<br>-<br>-<br>-<br>19:20<br>-	[429]TPKKKKRTGD[438] [9]<br>[289]TSKKRYESDPAFNKIGD-EDLEKCKKLKSE[317] [6.5]<br>[197]DIIKKASMAANHL-HLPEDKALISNPSNIIKL[227] [3.4]<br>[167]FGSVKEPSGDPQVLKTLIDIQFGCLLKNPSTT[198] [4.2]<br>[277]IYSIKRKISAMPLKDRI-ELLWKRTPET[303] [4.6]<br>[735]RETLKRIMED-LTCFLSKLGLPLKRAETW[762] [7.7]	276<br>313–324<br>-<br>-<br>-<br>1416, 1419	MMaV-N/4e-71/86<br>no hits<br>PeV1-P3/0.012/56<br>MIMV-M/2e-04/61<br>TaVCV-G/5e-90/90<br>MMV-L/0.0/94
Allium chinense virus 1	1	1<br>2<br>3<br>4<br>5<br>6<br>7<br>8	N<br>P<br>P'<br>P3<br>M<br>G*<br>P6*<br>L*	52.1<br>35.4<br>15.422<br>6.1<br>18.5<br>-<br>-<br>-	6.29<br>4.57<br>10.1<br>9.59<br>9.18<br>5.82<br>-<br>-	-<br>-<br>42–61, 82–107<br>-<br>-<br>492–514<br>-<br>-	-<br>-<br>-<br>-<br>-<br>20:21<br>-<br>-	-<br>-<br>-<br>-<br>-<br>-<br>-<br>-	308<br>-<br>-<br>-<br>119–126<br>-<br>-<br>53, 55, 418, 420	SCV-N/3e-140/96<br>ChYDaV-P/7e-60/100<br>RVCV-P'/0.001/34<br>ChYDaV-P3/3e-73/96<br>ChYDaV-M/6e-25/99<br>RVCV-G/6e-143/95<br>-<br>SCV-L/7e-70/100
Anthurium amnícola virus 1	1	1<br>2<br>3<br>4<br>5<br>6	N<br>P<br>P3<br>M<br>G<br>L	45.6<br>35.9<br>23.7<br>20.7<br>63.8<br>234.2	5.82<br>5.11<br>7.75<br>9.54<br>8.15<br>8.48	-<br>-<br>-<br>-<br>13–35, 40–59, 526–548<br>-	-<br>-<br>-<br>-<br>-<br>-	-<br>-<br>-<br>-<br>-<br>-	369<br>-<br>-<br>36, 126<br>-<br>88–89, 91<br>1259,1344, 1349	PMuMaV-N/1e-104/99<br>PMuMaV-P/2e-11/64<br>PMuMaV-P3/3e-23/76<br>PMuMaV-M/9e-33/98<br>PMuMaV-G/3e-76/96<br>PMuMaV-L/0.0/99

Asclepias syriaca virus 1	1	1	N	50	5.7	-	-	-	100, 102	WhIV4-N/1e-164/93
		2	P	33.8	4.58	-	-	-	-	WhIV4-P/8e-48/89
		3	P′	10.2	9.75	5–27, 32–51, 64–86	-	-	-	no hits
		4	P3	37.7	8.78	-	-	-	-	WhIV4-P3/4e-123/99
		5	M	19.6	9.16	-	-	-	128–134	WhIV-4-M/4e-23/91
		6	G	61.8	6.34	502–524	22:23	-	-	WhIV-4-G/6e-161/99
		7	P6	10.02	9.52	27–49	-	-	-	no hits
		8	L	241.6	8.86	-	-	-	-	WhIV4-L/0.0/99
Asclepias syriaca virus 2	1	1	N	51.8	8.73	-	-	^{413}IVARKRGHT-LGTIPDKPNTATKRSASE^{439} [5.2]	86	ApRVA-N/5e-146/98
		2	P	38.2	4.86	-	-	^{82}LHKNNFNQLSDVLLDGI VTFGKEIRLSTVP^{111} [4.1]	-	ApRVA-P/3e-11/69
		3	P3	39.3	8.62	-	-	^{70}ESYLNKRKIELSLK-NLFNVFSVEVDIKQITCIW^{102} [4.8]	-	ApRVA-P3/4e-36/95
		4	M	28.3	5.95	-	-	^{147}LDKDHGVIVSNIE-IFISPLTAEQFKASKL^{175} [5.5]	-	ApRVA-M/53-18/82
		5	G	73.5	6.43	560–582	25:26	^{250}RIDCHYKESSAILECPE-ITYSFPLHRLKKTPTCK^{283} [4.6]	-	ApRVA-G/5e-133/95
		6	L	233.4	7.23	-	-	^{790}EKMQKRGLPLKLEET-WISNRLLMYNKIMYL^{819} [5.8]	-	ApRVA-L/0.0/99
Bemisia tabaci -associated virus 1	1	1	N	50.2	8.67	-	-	-	61	CuCV1-N/0.0/100
		2	P	36.1	4.64	-	-	-	275	CuCV1-P/1e-148/96
		3	P3	21.6	9.35	-	-	-	-	CuCV1-P3/3e-96/100
		4	P4	5.99	10.29	5–22		-	10–16	CuCV1-P4/6e06/72
		5	M	23.8	8.96	-	-	-	-	CuCV1-M/2e-93/100
		6	G	65.7	7.46	22–39, 59–78, 538–557	-	-	-	CuCV1-G/0.0/100
		7	L	242.1	8.43	-	-	-	573, 577, 1423, 1465, 1983, 1985–1986, 1988, 1990	CuCV1-L/0.0/99
Brassica rapa virus 1		1	L	232.4	6.92	-	-	^{1594}FFKMNPSLIEDYGEEED-FKIKAEIKQFRRK^{1623} [5.6]	6, 11, 2004	RCaVV-L/0.0/99
	1	1	N	49.9	5.29	-	-	^{221}KLVERFEHFY-MANFPFEDFHPN-LESAKAIKSI^{252} [5.4]	373, 375, 378, 380	RCaVV-N/1e-43/72
	2	2	2	46.3	9.18	-	-	^{287}EPRL-KRKESPSPPILDVKKPKKSESK^{312} [6.8]	70, 190	no hits
		3	3	20.7	5.03	-	-	-	147	RCaVV-P3/2e-09/90
Cuscuta reflexa virus 1	1	1	N	50.8	6.72	-	-	^{440}EPTKKRKTPSL^{450} [8]	292	DYVV-N/0.0/97
		2	P	36.8	5.51	-	-	^{218}EQLRAK-VRLHYSDTEFDSFNDSKKMAL^{244} [4]	214–225	DYVV-P/8e-53/97
		3	P3	36.8	8.48	-	-		171, 173	

		4	M*	-	8.89	-	-	[114]RGRISESLKAV-VFPTKSILKNKNDAFF-MPWKFAA[147] [4]	-	DYVV-P3/1e-91/91
		5	G*	-	5.55	479–501	23:24	[200]RQKRVPRKRSKSPAYNTKKRGISKKRKPQ[228] [8.8]	-	GSPNuV-M/2e-77/100
		6	L*	-	-	-	-	[507]KVPSKVVTFVEDEMEDFKTPLRVPSAPKKRM[537] [5]	-	DYVV-G/0.0/92
								[288]EGVGQKRQHPSVQESIKRHMLSDRTHATRMRLS[320] [5.3]		DYVV-L/3e-71/98
Dioscorea composita virus 1	1	1	N	48.5	6.37	-	-	-	148	MYSV-N/5e-23/93
		2	P*	-	-	-	-	-	-	-
		3	P3*	-	-	-	-	-	-	-
		4	M	22.9	7.19	-	-	-	13	no hits
		5	G	66.2	6.51	539–556	-	-	-	PMuMaV-G/4e-04/85
		6	P6	8.54	11.18	23–45	-	-	-	no hits
		7	L*	-	-	-	-	-	37	RVR/0.0/95
Glehnia littoralis virus 1	1	1	N	46.4	5.6	-	-	-	264	TpVA-N/0.0/99
		2	P	35.2	5.47	-	-	-	-	TpVA-P/6e-136/99
		3	P′	10.3	10.4	13–35, 50–72	-	-	-	no hits
		4	P3	22.6	9.41	-	-	-	-	TpVA-P3/3e-114/100
		5	M	18.6	8.48	-	-	-	-	TpVA-M/8e-79/94
		6	G	60.9	6.5	503–525	21:22	-	-	TpVA-G/0.0/98
		7	P6	7.8	9.74	31–53	-	-	-	no hits
		8	L	238.3	8.23	-	-	-	95, 97–98, 100	TpVA-L/0.0/99
Gymnadenia densiflora virus 1	1	1	N	49	5.5	-	-	-	73, 80–86	LYMoV-N/6e-33/89
		2	P	34.6	6.49	-	-	-	124	SCV-P/0.002/42
		3	M	21.7	5.5	-	-	-	155	no hits
		4	L	237.7	7.81	-	-	-	1574	WhIV5-L/0.0/88
Lolium perenne virus 1	1	1	L	231.9	7.19	-	-	[108]RLVHKALKNRDIRI-PISTNDITEDLPDVKI-YHRW[141] [5.4]	-	AMVV1-L/0.0/100
	2	1	N	61.9	6.48	-	-	[134]VAPKKKKKRMRHF[146] [8.5]	-	AMVV1-N/2e-142/94
		2	2	43	5.84	-	-	[91]GKKKKPRLGRE[101] [8]	163, 165	no hits
		3	3	18.3	5.34	-	-	-	93–98	no hits
Lotus corniculatus virus 1	1	1	N	53.7	6.36	-	-	-	128–130 252, 262	LNYV-N/9e-77/96
		2	P	34.6	4.71	-	-	-	-	WhIV4-P/2e-25/57
		3	P′	11.8	10.6	30–47, 51–73	-	-	-	no hits
		4	P3	40.2	8.56	-	-	-	123–125	WhIV4-P3/2e-50/74
		5	M	19.8	8.75	-	-	-	-	CCyV1-M/0.001/53
		6	G	62.6	6.97	512–534	24:25	-	-	WhIV4-G/1e-101/98
		7	P6	6.65	9.57	21–43	-	-		no hits
		8	L*	-	8.31	-	-	-	832–833, 901, 905, 907, 909, 1484,1915, 1920, 1922	WhIV4-L/0.0/98
Melampyrum roseum virus 1	1	1	L	229.2	7.35	-	-	[107]IGDALKKRKID[117] [9.5]	-	RCaVV-L/0.0/96
	2	1	N	49.8	6.04	-	-	-	23	RCaVV-N/3e-17/91
		2	2	38.1	5.34	-	-	-	37	

		3	3	34.5	9.1	-	-	-	71–78	no hits
		4	4	22	5.42	-	-		81	no hits
										no hits
Nymphaea alba virus 1	1	1	N	47.4	5.8	-	-	-	-	TpVA-N/6e-115/95
		2	P	33.3	4.8	-	-	-	287	TpVA-P/1e-22/99
		3	P′	10	9.1	7–29, 33–52, 57–79	-	-	-	no hits
		4	P3	25.4	8.68	-	-	-	-	StrV1-P3/3e-32/69
		5	M	21.3	8.94	-	-	-	36	KePCyV-M/3e-11/50
		6	G	62.5	6.48	504–526	22:23	-	-	KePCyV-G/1e-146/93
		7	P6	8.3	9.78	32–54	-	-	-	no hits
		8	L	237.7	8.46	-	-	-	-	TpVA-L/0.0/98
Pelargonium radula virus 1	1	1	N	53.9	7.07	-	-	-	135	WhIV4-N/3e-121/98
		2	P	33.9	4.51	-	-	-	277, 281	WhIV4-P/4e-27/90
		3	P′	8.57	10.1	4–26, 33–55	-	-	-	no hits
		4	P3	39.1	8.63	-	-	-	-	WhIV4-P3/5e-73/94
		5	M	19.5	8.3	-	-	-	61	WhIV4-M/2e-06/85
		6	G*	-	6.04	20–42, 529–551	41:42	-	-	WhIV4-G/1e86/93
		7	P6	6.66	10.2	15–37	-	-	-	no hits
		8	L*	-	-	-	-	-	89, 91,112	WhIV4-L/0.0/97
Persicaria minor virus 1	1	1	N*	-	-	-	-	^{47}LFKIPGALNNCALIG-PPTSHQRAAKCQWCK^{76} [4.9]	-	GSPNuV-N/7e-131/97
		2	P*	-	-	-	-	^{51}NLFKAEGLTIPNETIS-VLGEVSLVCKKHGRE^{80} [3.5]	-	DYVV-P/4e-18/72
		3	P3*	-	-	-	-	-	-	DYVV-P3/3e-42/81
		4	M*	-	-	-	-	^{183}PEKKKRPRTS^{192} [8]	120–122, 124	GSPNuV-M/2e-63/96
		5	G*	-	-	-	-	^{202}RNPGWF-SKVGLTRSMLHS-QADIAGISSPRCRPIKK^{236} [3.7]	-	GSPNuV-G/6e-72/95
		6	L*	-	-	-	-	^{356}TAEKFKTGVLLSAYGDE DLLKRALKIQKL^{384} [7]	66, 73, 303–304	DYVV-L/4e-45/67
Phlox pilosa virus 1	1	1	6*	-	-	-	-	-	-	-
		2	L*	-	-	-	-	-	79, 81, 83	LBVaV-L/1e-154/97
	2	1	N	42.3	5.9	-	-	-	67, 69	LBVaV-N/3e-45/99
		2	2*	-	-	-	-	-	-	no hits
		3	3	31.7	8.74	-	-	-	236, 239–242, 244	LBVaV-P3/3e-50/97
		4	4*	-	-	-	-	-	25–29	no hits
		5	5*	-	-	-	-	-	-	no hits
Pinus flexilis virus 1	1	1	N	45.7	8.28	-	-		321	AMVV1-N/4e-18/86
		2	2	50.2	4.92	-	-	-	191, 193, 196, 198	no hits
		3	3	35.4	8.8	-	-	^{79}KVLKRVK-DEGEGSRQSPKDKGK-TRKNPFVI^{108} [5.9]	278, 280	
		4	4	25.2	5.32	-	-		168	LBVaV-P3/5e37/80

		5	L	232.6	7.54	-	-	232PRRPSSKMIQT-YARRGVNSASRATKIIKL250 [5.9]<br>-<br>-	26	no hits<br>LBVaV-L/0.0/97
Plectranthus aromaticus virus 1	1	1	N	50.2	8.13	-	-	421DNEAKRKAPISE-DETAKKKRPPP443 [11.5]	350	DYVV/0.0/97
		2	P	36.7	5.16	-	-	140RKKVSIAK-KIEGKDITLPP-SQESQSKDPGDKTIK173 [5.1]	73	DYVV-P/8e-93/97
		3	P3	36.1	8.03	-	-	178ERVLYKPNVVSWSNLHYPYYIPFYMEKKVRGI209 [3.4]	-	DYVV-P3/1e-160/100
		4	M	32	8.71	-	-	220RPKQRFNKRPGLSPSSA-PAKKILN243 [5.5]	150, 154	DYVV-M/8e-97/94
		5	G	65.9	5.91	500–522	-	538REKIV-KFNPDALEQFVPDNLVHPTAPKRRPQS569 [4.1]	-	DYVV-G/0.0/100
		6	L	238.1	8.26	-	-	1619DQTTSKRKRAG-GLFSYIKRARD1640 [9.8]	166, 1455	DYVV-L/0.0/100
Rhododendron delavayi virus 1	1	1	N	51.1	8.6	-	-	436RGKRPLEQEGGIP-KRPAMESVPVAPLPFST465 [4.6]	-	SYVV-N/9e-126/89
		2	P	38.3	5.19	-	-	227RDRCRKHYT-SELFESWDDAKKRTSI251 [8.8]	-	BCaRV-P/7e-30/97
		3	P3	36.9	8.16	-	-	230DHCKRLKISEEDSWN-LMQTMTHEDANKIM-DKT261 [4.1]	236	BCaRV-P3/2e-59/91
		4	M	31.2	5.77	-	-	225KSYLKHLLSRVGKKRGRDDSPYQLRKEKLSKT255 [7.4]	-	BCaRV-M/6e-29/79
		5	G	73	8.54	558–577	23:24	82KQACLNEQKETEITIAIM-KWDFKTDKIP109 [5.4]	-	DYVV-G/1e-132/96
		6	L	233	8.37	-	-	1656KKGKRKIGSLQELLNRGAAAKRIRVS1681 [9]	1458	BCaRV-L/0.0/95
Spinach virus 1	1	1	L*	231.3	7.34	-	-	1936KATKRNRLD1944 [5.5]	-	RCaVV-L/0.0/99
	2	1	N	50.6	6.15	-	-	-	292	RCaVV/4e-42/91
		2	2	50.5	5.59	-	-	5AIKRTSQATLQEGED-TRKSTRL26 [5]	377, 382	no hits
		3	3*	-	-	-	-	-	63	RCaVV-P3/8e-04/90
Suaeda salsa virus 1	1	1	N*	-	-	-	-	-	172, 175, 178	SCV-N/6e-107/97
		2	P*	-	4.72	-	-	-		RVCV-P/4e-46/82
		3	P′*	-	-	36–58	-	-	-	no hits
		4	P3	24.4	9.41	-	-	-	-	ChYDaV-P3/1e-55/85
		5	M	18.2	9.52	-	-	-	-	RVCV-M/8e-25/96
		6	G*	-	-	-	-	-	119–126	ChYDaV-G/2e-14/98
		7	P6*	-	-	-	-	-	-	no hits
		8	L*	-	-	-	-	-	294	RVCV-L/8e-119/80
Tagetes erecta virus 1	1	1	N	59.9	5.47	-	-	-	69, 72	RSMV-N/8e-42/66
		2	P	56.6	5.48	-	-	-		PpVE-P/1e-09/55
		3	P3	23.1	9.57	-	-	-	-	RVR-P3/3e-20/66
		4	M	21.1	8.84	-	-	-	-	MYSV-M/0.001/45
		5	L	238.8	7.04	-	-	-	-	MYSV-L/0.0/99

									123, 1957, 1959,1961	
Trachyspermum ammi virus 1	1	1	N	50.4	6.02	-	-	-	88–92	StrV1-N/4e-30/62
		2	P	36.6	7.64	-	-	-	131	no hits
		3	P3	26.5	8.43	-	-	-	-	StrV1-P3/0.002/49
		4	M	22.8	4.71	-	-	-	60, 62–63	CCyV1-M/2e-04/44
		5	L	236.8	7.26	-	-	-	-	WhIV5-L/0.0/96
Viola verecunda virus 1	1	1	N	55.8	5.3	-	-	^{427}PRPEKRKRADRE^{438} [10]	26, 176	OFV-N/2e-35/72
		2	P*	-	-	-	-	-	-	-
		3	P3	36.6	8.59	-	-	^{47}RDVVCK-GKFGPNLLRRWGNSTHHSVCVKEIKID^{79} [5.6]	-	ORF-P3/7e-05/61
		4	M*	-	-	-	-		-	-
		5	G	74.4	5.54	618–640	-	-	-	OFV-G/4e-05/47
	2	1	L*	-	-	-	-	^{31}KQTPEQAPADPDVRPERELKDEKMTEM^{57} [3.6] ^{1655}IHPKGKKRKMK^{1665} [11.5]	1466	ClCSV-L/1e-153/60

[a] ORF numbers are represented from 3′ to 5′ for genomic sense and correspond to those shown in Fig.1.
* Coding region not complete
\# The predicted NLS with the highest score is shown
TMHMM—Transmembrane domain.
NLS—Nuclear localization signal
NES—Leucine-rich nuclear export signalNames and abbreviations of viruses are listed in Supp. Table S1

Table S4. Size of each encoded protein of every publicly available alpha-, beta, and gamma nucleorhabdovirus, cytorhabdovirus, varicosavirus and dichorhavirus.

	Virus	N	X	P	P3	Y/P3/P4	P4	P5	P6	M	U	G	P9	P10	L	8
	AntAmnV1	**409**	**NA**	**314**	**NA**	**207**	**NA**	**NA**	**NA**	**176**	**NA**	**565**	**NA**	**NA**	**2047**	**NA**
	AscSyV1	**445**	**NA**	**304**	**NA**	**334**	**NA**	**NA**	**NA**	**176**	**NA**	**551**	**86**	**NA**	**2101**	**NA**
	BeTaV1	**447**	**NA**	**326**	**NA**	**187**	**NA**	**NA**	**NA**	**206**	**NA**	**579**	**NA**	**NA**	**2102**	**NA**
	GilV1	**412**	**NA**	**324**	**NA**	**201**	**48**	**NA**	**NA**	**167**	**NA**	**551**	**66**	**NA**	**2072**	**NA**
	GymDenV1	**444**	**NA**	**310**	**NA**	**NA**	**NA**	**NA**	**NA**	**189**	**NA**	**NA**	**NA**	**NA**	**2068**	**NA**
	NymAV1	**421**	**NA**	**308**	**NA**	**228**	**NA**	**NA**	**NA**	**195**	**NA**	**560**	**69**	**NA**	**2076**	**NA**
	TaEV1	**520**	**NA**	**505**	**NA**	**200**	**NA**	**NA**	**NA**	**187**	**NA**	**NA**	**NA**	**NA**	**2079**	**NA**
	TrAV1	**455**	**NA**	**343**	**NA**	**237**	**NA**	**NA**	**NA**	**200**	**NA**	**NA**	**NA**	**NA**	**2069**	**NA**
Cytorhabdovirus	ADV	482	NA	311	NA	240	NA	NA	NA	190	NA	564	65	NA	2085	NA
	BmV1	455	NA	291	NA	342	NA	NA	NA	169	NA	546	75	NA	2107	
	BYSMV	427	NA	295	NA	171	124	79	100	166	NA	478	51	NA	2056	NA
	CCyV1	460	NA	298	NA	347	NA	NA	NA	168	NA	575	NA	NA	2071	NA
	CBDaV	422	NA	280	NA	192	NA	NA	NA	170	NA	503	52	NA	2068	NA
	ChYDaV	342	NA	326	NA	241	NA	NA	NA	173	NA	584	67	NA	2116	
	CuCV1	455	NA	319	NA	244	52	NA	NA	210	NA	578	NA	NA	2103	NA
	KePCyV	423	NA	322	NA	208	NA	NA	NA	155	NA	566	58	NA	2092	
	LNYV	459	NA	300	NA	302	NA	NA	NA	177	NA	551	NA	NA	2068	NA
	LYMoV	452	NA	306	NA	311	NA	NA	NA	175	NA	548	NA	NA	2068	NA
	MaCyV	445	NA	298	NA	169	NA	NA	NA	167	NA	486	NA	NA	2066	NA
	MYSV	431	NA	292	NA	181	123	72	105	167	NA	476	51	NA	2056	NA
	NCMV	431	NA	286	NA	172	114	125	122	174	NA	483	NA	NA	2058	NA
	PMuMaV	414	NA	327	NA	214	NA	NA	NA	183	NA	575	NA	NA	2059	NA
	PpVE	451	NA	445	NA	189	26	28	NA	214	NA	519	NA	NA	2113	NA
	PeVA	450	NA	312	NA	222	NA	NA	NA	188	NA	560	NA	NA	2089	NA
	RVR	473	NA	381	NA	180	NA	NA	NA	181	NA	561	65	NA	2072	
	RSMV	491	NA	375	NA	177	NA	NA	NA	174	NA	536	66	NA	2066	NA
	RVCV	484	NA	340	NA	238	NA	NA	NA	197	NA	567	64	NA	2088	NA

	SCV	465	NA	329	NA	243	NA	NA	NA	185	NA	568	67	NA	2089	NA
	StrV1	421	NA	355	NA	224	NA	NA	NA	180	NA	551	69	74	2092	NA
	TpVA	452	NA	326	NA	201	NA	NA	NA	166	NA	553	67	NA	2073	NA
	TpVB	450	NA	311	NA	307	NA	NA	NA	172	NA	552	NA	NA	2068	NA
	TrARV1	456	NA	300	NA	226	NA	NA	NA	176	NA	553	NA	NA	2104	NA
	TYMaV	412	NA	345	NA	224	NA	NA	NA	195	NA	560	135	NA	2085	NA
	WhIV4	485	NA	301	NA	339	NA	NA	NA	172	NA	571	76	NA	2105	NA
	WhIV5	452	NA	302	NA	225	NA	NA	NA	187	NA	550	NA	NA	2098	NA
	WhIV6	455	NA	299	NA	234	NA	NA	NA	173	NA	565	66	NA	2079	NA
	YmCaV	422	NA	386	NA	187	67	NA	NA	217	NA	602	NA	NA	2134	NA
	YmVA	488	NA	516	61	240	NA	NA	NA	202	NA	155	NA	NA	2243	141
Alphanucleorhabdovirus	ATV1	452	NA	335	NA	287	NA	NA	NA	257	NA	597	NA	NA	1937	NA
	CYDV	473	79	279	NA	287	NA	NA	NA	254	NA	609	NA	NA	1944	NA
	EMDV	476	97	294	NA	287	NA	NA	NA	251	NA	615	NA	NA	1946	NA
	JYBaV	470	91	279	NA	286	NA	NA	NA	254	NA	605	NA	NA	1946	
	MIMV	445	NA	270	NA	282	NA	NA	NA	233	NA	594	NA	NA	1926	NA
	MMV	447	NA	269	NA	286	NA	NA	NA	235	NA	591	NA	NA	1922	NA
	MMaV	455	NA	283	NA	288	NA	NA	NA	234	NA	589	NA	NA	1925	NA
	PeV1	468	NA	293	NA	278	NA	NA	NA	247	NA	591	NA	NA	1935	NA
	PhCMoV	476	101	294	NA	287	NA	NA	NA	251	NA	624	NA	NA	1946	NA
	PYDV	472	86	280	NA	285	NA	NA	NA	253	NA	607	NA	NA	1931	NA
	RYSV	513	NA	322	NA	294	NA	NA	NA	280	NA	692	93	NA	1964	NA
	TaVCV	502	NA	271	NA	287	NA	NA	NA	235	NA	588	NA	NA	1928	NA
	WYSV	544	NA	349	NA	335	NA	NA	NA	270	NA	661	NA	NA	1965	NA
Betanucleorhabdovirus	AscSyV2	459	NA	337	NA	343	NA	NA	NA	250	NA	643	NA	NA	2023	NA
	PleArV1	450	NA	332	NA	321	NA	NA	NA	286	NA	582	NA	NA	2085	NA
	RhoDeV1	464	NA	338	NA	326	NA	NA	NA	279	NA	636	NA	NA	2016	NA
	AaNV	443	NA	363	NA	322	NA	NA	NA	277	113	629	NA	NA	2038	NA
	ApRVA	450	NA	317	NA	369	NA	NA	NA	280	162	649	NA	NA	2057	NA
	BFTaV	462	NA	344	NA	325	NA	NA	NA	294	NA	637	NA	NA	2104	NA
	BCaRV	468	NA	326	NA	322	NA	NA	NA	276	NA	641	NA	NA	2106	NA
	BmV2	450	NA	319	NA	322	NA	NA	NA	286	NA	630	NA	NA	2102	
	CdVCV1	469	NA	347	NA	322	NA	NA	NA	302	NA	640	NA	NA	2095	NA
	DYVV	450	NA	327	NA	321	NA	NA	NA	284	NA	630	NA	NA	2106	NA
	GSPNuV	460	NA	360	NA	332	NA	NA	NA	267	NA	635	NA	NA	2118	NA
	SYNV	475	NA	345	NA	324	NA	NA	NA	286	NA	632	NA	NA	2116	NA
	SYVV	468	NA	390	NA	321	NA	NA	NA	284	NA	638	NA	NA	2106	NA
Gammanucleor	MFSV	462	NA	338	93	327	NA	NA	NA	246	NA	596	NA	NA	1944	NA

		N	2	3	4	5	6	L
Varicosavirus	BrRV1	435	411	180	NA	NA	NA	2019
	LoPV1	533	379	161	NA	NA	NA	2029
	MelRoV1	443	350	304	193	NA	NA	2003
	PiFleV1	405	447	318	219	NA	NA	2049
	AMMV1	501	327	152	NA	NA	42	2031
	LBVaV	397	363	290	164	368	40	2040
	RCaVV	429	427	179	NA	NA	NA	2018

		N	P	P3	M	G	L
Dichorhavirus	CiCSV	448	238	328	183	542	1874
	CILV-N	450	236	330	186	538	1879
	ClCSV	445	238	328	183	533	1874
	CoRSV	446	238	328	183	534	1864
	OFV	450	237	370	183	580	1877

The protein sizes of those viruses which complete coding region was assembled in our study are shown in bold and highlighted in light grey, while in yellow are highlighted the biggest and smallest sizes found among the alpha- and betanucleorhabdovirus, cytorhabdovirus, varicosavirus and dichorhavirus sequences. Orange indicates accessory genes, light blue when not applicable.
